# *ANO10* Function in Health and Disease

**DOI:** 10.1007/s12311-022-01395-3

**Published:** 2022-06-01

**Authors:** Androniki Chrysanthou, Antonis Ververis, Kyproula Christodoulou

**Affiliations:** 1 Neurogenetics Department, The Cyprus Institute of Neurology and Genetics, Iroon Avenue 6, Agios Dometios 2371, Nicosia, Cyprus; 2 The Cyprus School of Molecular Medicine, The Cyprus Institute of Neurology and Genetics, Iroon Avenue 6, Agios Dometios 2371, Nicosia, Cyprus

**Keywords:** Ataxia, SCAR10, Anoctamin, ANO10

## Abstract

Anoctamin 10 (ANO10), also known as TMEM16K, is a transmembrane protein and member of the anoctamin family characterized by functional duality. Anoctamins manifest ion channel and phospholipid scrambling activities and are involved in many physiological processes such as cell division, migration, apoptosis, cell signalling, and developmental processes. Several diseases, including neurological, muscle, blood disorders, and cancer, have been associated with the anoctamin family proteins. ANO10, which is the main focus of the present review, exhibits both scrambling and chloride channel activity; calcium availability is necessary for protein activation in either case. Additional processes implicating ANO10 include endosomal sorting, spindle assembly, and calcium signalling. Dysregulation of calcium signalling in Purkinje cells due to ANO10 defects is proposed as the main mechanism leading to spinocerebellar ataxia autosomal recessive type 10 (SCAR10), a rare, slowly progressive spinocerebellar ataxia. Regulation of the endolysosomal pathway is an additional ANO10 function linked to SCAR10 aetiology. Further functional investigation is essential to unravel the ANO10 mechanism of action and involvement in disease development.

**Supplementary Information:**

The online version contains supplementary material available at 10.1007/s12311-022-01395-3.

## Introduction

The anoctamin family (alternatively named TMEM16) consists of ten transmembrane proteins (ANO1-10) characterized by functional diversity and differential tissue expression [1]. The anoctamins were previously considered to have eight transmembrane domains and chloride channel function. Further studies [2–4] revealed that they consist of two monomers, having ten transmembrane domains each, and are involved in different processes. Human anoctamin proteins exhibit calcium-activated chloride channel (CaCC) activity (ANO1 and -2), phospholipid scrambling activity (ANO3, -4, -7, -9), or dual activity (ANO5 and -6). However, the function remains not fully elucidated for the remaining ANO family members (ANO 8 and -10) [5]. Anoctamins are involved in many physiological processes including cell proliferation, migration, apoptosis [6], epithelial secretion, blood clotting, neuronal and cardiac cell excitability, sensory transduction [1, 7], murine cephalic development [8], and embryogenesis [9]. Anoctamins have been linked to several diseases. ANO5 was the first anoctamin to be implicated in a genetic disorder, gnathodiaphyseal dysplasia, a rare, autosomal dominant (AD) bone dysplasia. *ANO5* mutations can lead to muscle disorders as well; muscular dystrophy (LGMD2L) and Miyoshi muscular dystrophy 3, both inherited in an autosomal recessive (AR) pattern. ANO6, the best-characterized scramblase of the anoctamin family, is mutated in Scott syndrome, a rare hemorrhagic disorder (AR) [1]. *ANO10* variants are known to cause spinocerebellar ataxia type 10 (AR) [10], while *ANO3* variants are associated with craniocervical dystonia (AD) [11]. Furthermore, upregulation of anoctamins has been associated with several types of human cancers; ANO1 is upregulated in head and neck squamous cell carcinomas [10], gastrointestinal stromal tumours, and breast cancer [11]. At the same time, it also contributes to the progression of autosomal dominant polycystic kidney disease (ADPKD), while ANO7 upregulation is linked to prostate cancer [11]. Given the variety of biological processes and diseases that anoctamins are involved in, it is crucial to understand their physiology and function.

This manuscript is a systematic review discussing the normal function of *ANO10* (*TMEM16K*) and its involvement in disease. Particular focus is given to the implication of *ANO10* in autosomal recessive spinocerebellar ataxia type 10 (SCAR10). This is the first systematic review summarizing all the known *ANO10* variants associated with SCAR10 and their clinical manifestation, hence facilitating diagnosis and disease prognosis. This review also discusses the proposed mechanisms of *ANO10* inducing SCAR10.

Studies have shown that ANO10 manifests both Ca^2+^–dependent chloride channel activity and phospholipid scrambling activities [12–15]. ANO10 scrambling activity helps maintain lipid distribution in the endoplasmic reticulum (ER) membrane [16]. Endosomal sorting [17], spindle assembly [18], Ca^2+^ signalling, apoptotic cell death, and changes in cell volume [19] are other processes that have been linked to ANO10. *ANO10* pathogenic variants have been associated with SCAR10, a rare, slowly progressive neurodegenerative disorder [20]. The exact mechanism of pathogenesis remains unclear; however, irregular Ca^2+^ signalling in Purkinje cells caused by *ANO10* pathogenic variants is a proposed mechanism [20–22]. So far, 94 *ANO10* variants were reported in ClinVar. Forty-one *ANO10* variants have been associated with SCAR10 (NM_018075.5), according to the literature and ClinVar database [23]. Regarding the variants identified from the literature (35), six have been classified as pathogenic, four as pathogenic/likely pathogenic, three as likely pathogenic and three as variants of uncertain significance, while the remaining nineteen *ANO10* variants have not been reported to ClinVar. Five pathogenic and one likely pathogenic *ANO10* variants have also been reported to ClinVar but not in bibliography (Table 1). Furthermore, variants in anoctamin10 have been associated with immunological defects [24], and mitochondrial dysfunction [25]. Understanding the physiological role and regulation of *ANO10* could yield a potential therapeutic target for these conditions.

**Table 1:** SCAR10 – associated variants in the *ANO10* gene, that are reported in ClinVar and/or bibliography

*ANO10* variation (NM_018075.5)	Genomic location (GRCh38)	Clinical significance (according to ClinVar)
SCAR-10 associated *ANO10* variants reported in literature		
c.337 + 1G > A	3:43,600,383	Pathogenic
c.1150_1151del [p.Leu384fs]	3:43,576,703–43,576,704	Pathogenic
c.306C > A [p.Tyr102*]	3:43,600,415	Pathogenic
c.1529T>G [p.Leu510Arg]	3:43,555,417	Pathogenic
c.1604del [p.Leu535*]	3:43,555,342	Pathogenic
c.1476 + 1G > T	3:43,561,219	Pathogenic
c.1009T>G [p.Phe337Val]	3:43,576,845	Pathogenic/likely pathogenic
c.132dup [p.Asp45fs]	3:43,605,720–43,605,721	Pathogenic/likely pathogenic
c.96del [p.Glu33fs]	3:43,605,757	Pathogenic/likely pathogenic
c.289del [p.Thr96_Met97ins*]	3:43,600,432	Pathogenic/likely pathogenic
c.1537T>C [p.Cys513Arg]	3:43,555,409	Likely pathogenic
c.1088_1093delinsTCCTT [p.Ser363Ilefs*7]	3:43,576,766	Likely pathogenic
c.139 + 1G > T	3:43,605,713	Likely pathogenic
c.1537 T>C [p.Cys513Arg]	3:43,577,238	Uncertain
c.512T>C [p.Phe171Ser]	3:43,580,433	Uncertain
c.1843G > A [p.Asp615Asn]	3: 43,432,682	Uncertain
c.518delT [p.Leu173Argfs*7]	3:43,580,428	Not reported
c.1418delA [p.Asp473Alafs*36]	3:43,561,278	Not reported
c.(1797 + 1_17981)_(1913 + 1_1914-1)del [p.His600_Glu638del]	3:43,549,719–43,366,975	Not reported
c.1291C > T [p.Gln431*]	3:43,565,655	Not reported
c.1558dupG [p.Ala520Glyfs*7]	3:43,555,388	Not reported
c.1244C > G [p.Ser415*]	3:43,565,702	Not reported
c.123_124insA [p.Asp45Argfs*9]	3:43,605,720	Not reported
c.132_133insT [p.Asp45Argfs53*]	3:43,605,720	Not reported
c.1315G > T [p.Glu382*]	3:43,561,381	Not reported
c.1668 + 1G > A	3:43,555,277	Not reported
c.609C > G [p.Tyr203*]	3: 43,577,246	Not reported
c.1214delT [p.Leu405*]	3:43,574,813	Not reported
c.493_494dup [p.Ile166Alafs*3]	3:43,580,449–43,580,450	Not reported
c.1A > T	3:43,605,852	Not reported
c.1219-1G > T	3:43,565,728	Not reported
c.1664G > C [p.Trp555Ser]	3:43,555,282	Not reported
c.815G > C [p.Trp272Ser]	3:43,577,039	Not reported
del ex.12	3:43,432,727–43,432,611	Not reported
c.685G > T [p.Gly229Trp]	3: 43,577,169	Not reported
SCAR-10 associated *ANO10* variants reported in ClinVar only		
c.1551dup [p.Ala518fs]	3:43,555,394–43,555,395	Pathogenic
c.1025G > A [p.Trp342*]	3:43,576,829	Pathogenic
c.512T>C [p.Phe171Ser]	3:43,600,515	Pathogenic
c.1669-2A > T	3:43,549,850	Pathogenic
c.1163-9A > G	3:43,574,873	Pathogenic
c.473-2A > T	3:43,580,474	Likely pathogenic

## Methods

### Literature Search Strategy

We searched three electronic databases on the 8^th^ of February 2022 to complete the present study: PubMed, MEDLINE complete (EBSCOhost), and Academic Search Ultimate (EBSCOhost). The following search terms were used: Anoctamin10 OR anoctamin-10 OR ano10 OR ano10a OR ano10b OR TMEM16K OR (Drosophila AND Axs) OR (Drosophila AND “Aberrant X Segregation”) OR (“Oryza Sativa” AND Os01g0706700) OR (“Arapidopsis thaliana” AND AT1G73020) OR (“Anopheles gambiae” AND AgaP_AGAP009776) OR (Anoctamin10 OR anoctamin-10 OR ano10 OR TMEM16K AND (ataxia OR “spinocerebellar ataxia” OR SCA OR ARCA OR “hereditary cerebellar ataxia” OR HCA OR disease)). We excluded any record type other than a journal article, case report, observational study, or short communication/report. Duplicates were removed using the Mendeley Desktop software, leaving 68 records for further assessment. We also excluded records not in the English language, records with no full-text accessibility, and records in which none of the search terms was mentioned in the main text. Three independent reviewers manually evaluated the titles and abstracts of the remaining records, resulting in 53 publications. Supplementary Fig. 1 summarizes the study flow diagram. This systematic review was reported according to the PRISMA guidelines [26].

## Results

### ANO10 Localization and Function

The *ANO10* gene spans 2734 kb at chromosome 3p22.1 and contains 13 exons, 12 of which code for 660 amino acids [20, 27]. ANO10 is highly expressed in the human brain, mainly in the frontal and occipital cortex and cerebellum. The expression is higher in the brain of adults than fetuses, implying that except for its role in brain development, ANO10 is also involved in mature brain functions [20]. ANO10 is also highly expressed in the primary cilia of retinal pigment epithelium (RPE) cells [28] and is primarily found in the ER membrane [13, 24]. We did not find any reports or data comparing the levels of *ANO10* expression in the brain and the retina. Different lines of evidence support that ANO10 protein functions as an ion channel or a phospholipid scramblase, depending on calcium availability for activation in either case [29].

Early studies have suggested that ANO10 does not exhibit channel activity. Manoury et al. (2010) reported that ANO10 downregulation had no effects on the generation of CaCC currents in rat pulmonary artery smooth muscle cells. Rat ANO10 is less than 20% similar in primary structure with ANO1, which is a well-established CaCC. The fact that the pore-forming part of ANO1 (25 amino acids) is missing from ANO10 and that ANO10 is highly expressed even in tissues with no CaCC activity further support the view that ANO10 shows no channel activity [30]. Additional studies revealed that ANO10 has no effect in the production of CaCC currents [31] or in volume-induced currents (when intracellular calcium is absent) [32] when overexpressed in HEK293 cells. In addition, ANO10 was not found to be involved in CaCC currents activation in the choroid plexus [33]. However, several other studies contrast the above findings, indicating that ANO10 exhibits chloride channel activity. Tian et al. (2012) and Hammer et al. (2015) reported that ANO10 could produce Cl^−^ currents in HEK293 cells after ATP stimulation and subsequent intracellular Ca^2+^ increase [14] or hypotonic solution activation [24], respectively. Cl^−^ currents are also generated when ANO10 – expressing lymphocytes, macrophages, and *Xenopus* oocytes are exposed to a hypotonic solution [24]. Schreiber et al. (2010) showed that whole-cell Cl^−^ currents could be produced by ANO10 after intracellular Ca24 increase in Fisher rat thyroid (FRT) cells [15]. Moreover, ANO10 is capable of producing Cl^−^ currents after purinergic stimulation even when targeted to the plasma membrane of HEK293 cells [12]. Other studies support the CaCC function of ANO10 in the mouse intestine [34] and airways [35]. Notably, ANO10 is not Cl^−^-selective according to Bushell et al. (2019) [13]. Tian et al. (2012) found that ANO10 is also permeable to cations [14] while Viitanen et al. (2013) suggested that it may be involved in iodide transport in thyroid cells [36].

A second function associated with ANO10 is the scramblase activity. Two independent studies showed that ANO10 scrambles phospholipids in intracellular membranes. Tsuji et al. (2019) showed that ANO10 rearranges the phosphatidylserine (PS) of ER and nuclear membrane in the presence of calcium [16]. Bushell et al. (2019) suggested that ANO10 scrambling activity depends on calcium availability and membrane thickness, i.e. it requires calcium and short-chain lipids for optimal activity. Remarkably, ANO10 was found to transport PS relatively slower than other phospholipids, suggesting that lipid composition is another factor affecting the scrambling activity. However, this factor does not probably affect scrambling in ER since its content in PS is low. Crystallization methods showed that ANO10 is a symmetrical homodimer. The transmembrane domain of each monomer has ten transmembrane subunits, which are α helices (TM1–TM10). The TM3–TM7 helices form a groove that acts as the lipid transfer pathway. Probably, the lipids-groove interaction is achieved due to the charged and hydrogen bonding amino acids lining the groove. ANO10 has three Ca^2+^-binding sites, two formed by TM6–TM8 α helices and one formed by TM10 and the cytoplasmic domain α10 [13]. An earlier study showed that the Glu448, Glu529, and Asp533 residues are necessary for calcium binding [37]. Serine at position 363, which is conserved among known scramblases, is probably also essential for the scrambling activity. The ANO10 groove is open in the presence of calcium, allowing ANO10 activation and scrambling activity, and closed when calcium is absent. Although Ca^2+^ ions are necessary for ANO10 activity, residual scrambling activity still occurs in their absence, suggesting the existence of an additional open groove conformation in calcium-free conditions. However, the changes in the ANO10 structure occurring from the open to the closed groove state are not attributed to differences in calcium binding [13].

Ca^2+^ signalling is involved in ion transport and apoptotic cell death. Loss of ANO10 prevented the activation of whole-cell currents and decreased the apoptotic rate in intestinal epithelial cells, indicating the importance of ANO10 in calcium-mediated ion transport and apoptosis [19]. *ANO10* involvement in Ca^2+^ signalling was also indicated when *ANO10* knockdown resulted in strong attenuation of ATP-induced Ca^2+^ signals in mouse renal proximal tubular epithelial cells [38]. In addition, the importance of ANO10 in apoptosis was further confirmed by the finding that transdifferentiation of the short-lived monocytes into the long-lived macrophages was accompanied by ANO10 loss, in parallel with caspase-3 activity loss. Furthermore, ANO10 loss in cisplatin-resistant FRT cells led to compromised apoptotic volume decrease (AVD) and regulatory volume decrease (RVD) [19].

ANO10 was recently proposed to be involved in endosomal sorting. Through binding with Rab7 of endosomes and phosphatidylinositols of endolysosomes, ANO10 can regulate the distribution of cargo molecules in the cell. Mice lacking ANO10 showed defective endosomal retrograde trafficking, rescued by the wild type protein, and impairments during the late endolysosomal pathway. Interestingly, the scrambling activity of ANO10 appears to be important for the function of endosomal sorting [17].

Apart from its ER localization, ANO10 was also found to be strongly associated with acetylated tubulin of spindles in mouse macrophages [19], similar to the *ANO10* ortholog (Axs) in Drosophila. Axs shows colocalization with the ER of the oocyte nucleus before meiotic spindle assembly and is associated with the spindle microtubules during spindle assembly. Axs defects result in abnormal spindle formation and chromosome segregation [18]. These findings may indicate an ANO10 implication in spindle formation and progression of meiosis, similar to the Axs.

### ANO10 and Disease

#### ANO10 and Association with SCAR10

Pathogenic variants in the *ANO10* gene are known to be associated with SCAR10; Vermeer et al. (2010) were the first to identify *ANO10* alterations in affected siblings of three different families [20]. SCAR10 (OMIM number #613728) [39] belongs to a heterogeneous group of neurological disorders known as ARCAs (Autosomal Recessive Cerebellar Ataxias), which belong to the broader group of inherited ataxias [40]. In contrast with the early onset (< 20 years) observed in the majority of ARCAs [40], the age of onset in SCAR10 is highly variable (range = 6–45 years) [41]. According to the Orphanet portal, the estimated prevalence is < 1/10^6^ [42]. SCAR10 common features include gait, limb and trunk ataxia, dysarthria, severe cerebellar atrophy, and ocular movement impairments, such as horizontal, vertical, downbeat nystagmus, and/or hypermetric saccades [39, 42]. Additional features, which vary among patients, include cognitive impairment [43], seizures, intellectual disability, low coenzyme Q10 (CoQ10) levels in muscle [44], and lower motor neuron involvement [20].

The absence of neuropathy from patients with *ANO10* pathogenic variants led to the additional nomenclature of the SCAR10 disease as ARCA3, and its categorization in the group of ARCAs without neuropathy alongside ARCA1 and ARCA2 [41]. ARCA1, which is inherited in an autosomal recessive pattern [45], is associated with *SYNE1* mutations [46] and its main clinical features include adult-onset (17–46 years), gait and limb ataxia, dysarthria, dysmetria, mild oculomotor abnormalities, and cerebellar atrophy [47]. ARCA2 is a childhood—or adolescence—onset ataxia disorder (1.5–19 years) caused by biallelic *ADCK3* mutations. It is characterized by gait ataxia, exercise intolerance, intellectual disability, low CoQ10 levels, cerebellar atrophy, and absence of neuropathy, while the disease progression is usually very slow or stable [48]. Considering the phenotypic overlap of ARCAs, ARCA3 should be identified in cases of progressive cerebellar ataxia with cerebellar atrophy, absence of polyneuropathy, and regardless of the onset age [41]. The International Parkinson and Movement Disorder Society Task Force for Nomenclature of Genetic Movement Disorders proposed a different nomenclature, ATX–ANO10. ATX prefix designates ataxia as the primary disease feature and distinguishes this genetic entity from other diseases with a similar phenotype [49].

Three siblings of a Dutch consanguineous family were the first patients described with SCAR10. Homozygosity mapping and targeted next-generation sequencing analysis revealed a homozygous *ANO10* variant (c.1529T>G [p.Leu510Arg]) in all affected individuals. Subsequent direct examination of the *ANO10* gene by Sanger sequencing led to the identification of three additional variants in other patients: c.1476+1G>T and c.1604del [p.Leu535*] in two French siblings and c.1150_1151del [p.Leu384fs] in three Romani siblings from Serbia [20]. The c.1150_1151del variant was found to be a founder mutation in the Romani population, causing a more severe phenotype exhibiting earlier onset and intellectual decline [50]. This variant showed a seemingly dominant inheritance (also known as pseudo-dominant inheritance), since several individuals were affected in successive generations. However, all the patients were homozygous for the specific *ANO10* variant. The unique clinical manifestation of c.1150_1151del carriers is probably attributed to genetic modifiers specific to the Romani population rather than to the nature of this variant [51].

The c.132dupA [p.Asp45fs] variant is the most frequent *ANO10* variant causing SCAR10 [43]. The clinical examination of compound heterozygote patients carrying the c.132dupA variant led to the expansion of the SCAR10 phenotype. Executive and attention impairments, seizures, and a porencephalic cyst observed in a single patient were added to the SCAR10 clinical presentation [44]. Though rarely found in a homozygous state, *ANO10* c.132dupA is associated with cognitive impairment, ranging from mild to severe [45, 52, 53]. Three siblings from a consanguineous family carrying the homozygous c.132dupA variant demonstrated significant deficits in multiple cognitive domains. One of the siblings showed impairments exclusively in executive functions, whereas the other two siblings appeared with more impaired performance. In particular, they showed pronounced cognitive and motor deficits, memory problems, and deteriorated visuoperceptual and visuoconstructive abilities, indicating widespread cerebral dysfunction. Interestingly, CoQ10 plasma levels were lower in the patients exhibiting the more severe phenotype [43]. Low levels of CoQ10 in muscle, plasma, or cerebrospinal fluid were also found in other patients carrying the homozygous c.132dupA variant [22]. CoQ10 supplementation was beneficial for some patients, improving their mobility, cognition or fatigue symptoms. However, further investigation is needed to elucidate CoQ10 contribution to SCAR10 pathology and its therapeutic potential [22, 44, 53]. Additional phenotypic features described in patients carrying the c.132dupA variant in homozygosity include telangiectasia of ocular vessels and bladder dysfunction [53]. Unusually, a patient with SCAR10 phenotype, homozygous for the c.132dupA variant, was presented with mild axonal neuropathy 43]. However, the absence of neuropathy from other patients carrying the same variant, and SCAR10 categorization in ARCAs without neuropathy [41] suggest that this is a coincidental finding, unrelated to SCAR10.

One of the patients presented with cerebellar ataxia and CoQ10 deficiency (compound heterozygous for the c.132_133insT [p.Asp45Argfs*53] and c.1843G>A [p.Asp615Asn] variants) also showed respiratory chain deficiency. This defect is usually found in mitochondrial disease. This finding suggests an association between SCAR10 and mitochondrial dysfunction, maybe due to abnormal ANO10-mediated calcium metabolism [25]. The Asp615Asn variant lies in the TM10-α10 Ca^2+^-binding site of ANO10. Experiments by Bushell et al. [13] revealed that this variant showed no effects on lipid scrambling under calcium-free or saturating calcium conditions [13]. However, Le et al. (2020) found that the ANO1 Asp884Asn variant, which corresponds to ANO10 Asp615Asn, significantly decreased ANO1 calcium sensitivity, implying that ANO10 Asp615Asn might also abate ANO10 calcium sensitivity and channel activation [54].

Nanetti et al. (2019) described eight SCAR10 diagnosed patients presenting an adult-onset, slowly progressive cerebellar syndrome with pyramidal signs. Most of the patients displayed executive, linguistic, and visuospatial dysfunctions, despite their normal Mini-Mental State Examination (MMSE) scores. All patients had difficulties performing or even failed cognitive tests, such as the Symbol Digit Modalities Test (SDMT) and Rey–Osterrieth Complex Figure (ROCF) test. Giant sensory evoked potentials (SEPs) were observed in SCAR10 patients for the first time in this study, indicating possible sensory and motor cortex degeneration [55].

A splice variant in homozygosity (c.1219-1G>T) was found in a patient exhibiting a distinct phenotype. Dysmetria, kinetic tremor, and tendon hyperreflexia in four limbs, and a steady postural tremor in upper limbs were observed in addition to ocular and speech abnormalities and cortical cerebellar atrophy. Interestingly, the transcranial cerebello-cerebral direct current stimulation (tCCDCS) procedure was noticeably beneficial for the postural tremor reduction [21].

Patients diagnosed with SCAR10 were reported outside Europe, as well. The first non-European patient reported with an *ANO10* variant was a Japanese patient homozygous for the nonsense variant c.609C>G [p.Tyr203*] [56]. Two additional Japanese patients were diagnosed with SCAR10, one homozygous for the c.493_494dup [p.Ile166Alafs*3] *ANO10* variant [57] and another homozygous for the c.616delG [p.Glu206Lysfs*17] variant [58]. A Chinese patient was also found to carry a homozygous nonsense mutation (c.1244C>G [p.Ser415*]). Furthermore, *ANO10* variants (c.132dupA [p.Asp45fs] and c.1244C>G [p.Ser415*]) were found to be the underlying cause of ataxia in two Korean siblings [60]. The Asian patients described, all harboring truncating mutations, had late disease onset (> 35 years old), in contrast with the earlier onset (6–30 years old) associated with the truncating c.1150_1151del [p.Leu384fs] variant. The exact cause of the difference in SCAR10 prevalence between Europe and Asia remains unknown [58]. No mutational hot spots exist in *ANO10*, since variants are found across the whole gene [55]. However, the presence of missense or in-frame variants either in homozygosity or associated with a truncating variant result in a milder phenotype [41].

**Fig. 1:**
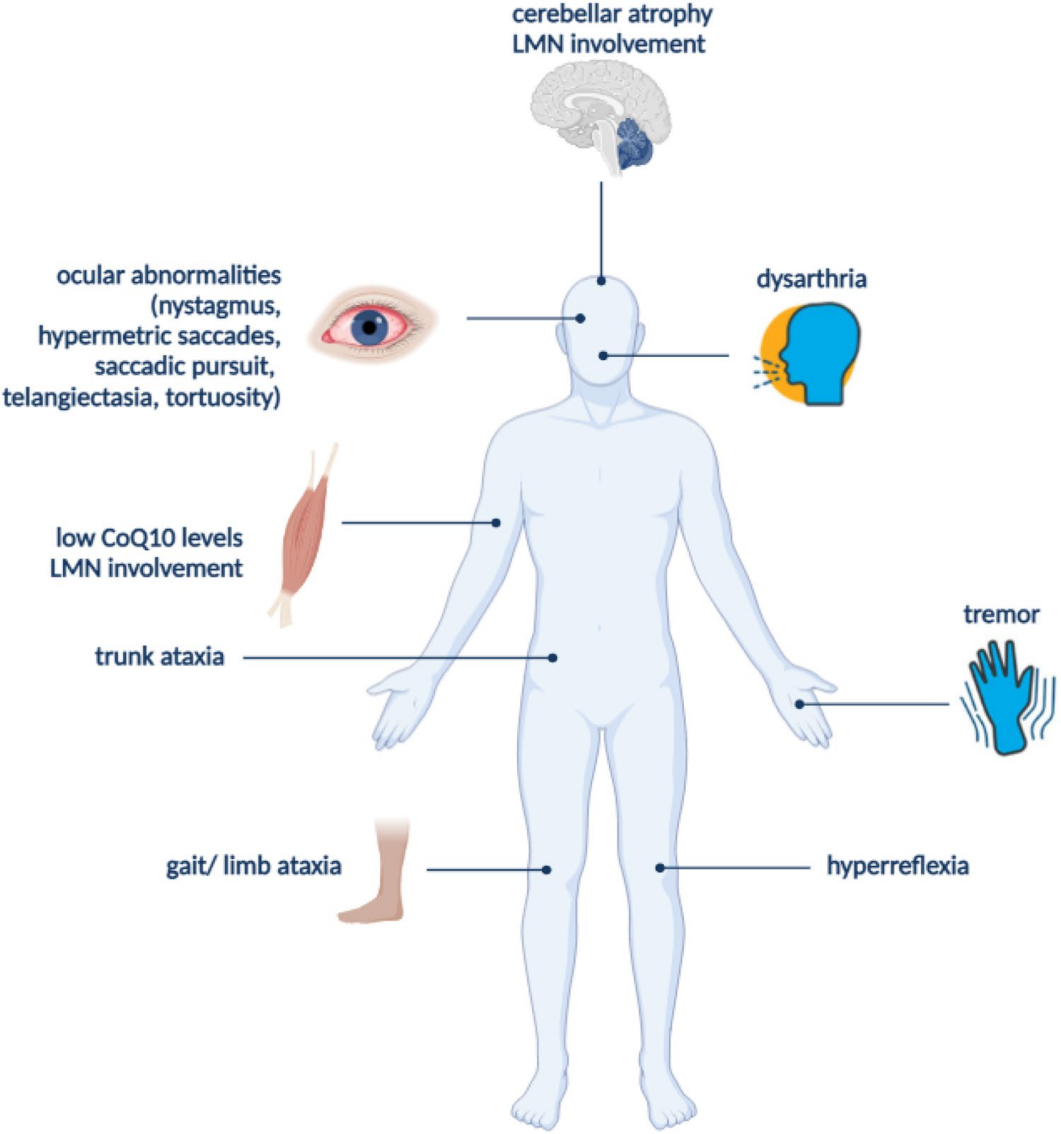
Main phenotypic features of SCAR10. Created with BioRender.com

All published to date cases of SCAR10 (71) are listed in Table 2. Their phenotypic features, if available, are summarized in the table. Brain MRI, performed on 55 patients, revealed cerebellar atrophy in all 55/55 (100%) patients, with five patients showing cortical atrophy and one patient slight brainstem atrophy, in addition to cerebellar atrophy. Cognitive evaluations, available for 51 patients, showed that 32/51 (62.7%) patients presented cognitive dysfunctions, while one patient had a low MMSE score (MMSE score = 19–29). The majority (61/71) of the patients (85.9%) exhibited oculomotor abnormalities, including 17 patients with downbeat nystagmus in a total of 58 patients with nystagmus (29.3%). Electromyographic findings were available for 35 patients; 16 (45.7%) showed motor neuron involvement and one mild axonal sensory neuropathy. The Scale for Assessment and Rating of Ataxia (SARA) was employed to evaluate the disease severity in 24 patients. The SARA score ranged from 5 to 24 (mean SARA score = 13.65). The average patient age at onset was 28.6 years (range = 6–53 years), while the average age at examination was 48.7 years (range = 23–70). Interestingly, the average onset age in the Romani population was lower than 28.6 years (17.7). Figure 1 summarizes the main clinical features of SCAR10.

**Table 2:** Phenotypic features of all reported to date patients carrying SCAR10—associated variants in the *ANO10* gene

Sex	Age at onset	Age at testing	Family history	ANO10 variant	Pathogenicity (according to ClinVar)	Phenotype	SARA score	COG	Brain MRI	Epileptic seizures	EMG
Aida et al. (2022) [58]											
M	36	39	No	c.616delG [p.Glu206Lysfs*17]	UN	Limb ataxia dysarthria downbeat nystagmus increased DTR LL spasticity	24	Mild cognitive decline	Marked cerebellar atrophy	No	Normal
Bogdanova-Mihaylova et al. (2020) [52]											
F	43	61	Yes	c.132dupA [p.Asp45fs] (HOM)	P/LP	Ataxia UMN features telangiectasia	NA	Yes	NA	NA	NA
F	9	70	Yes	c.132dupA [p.Asp45fs], c.96del [p.Glu33fs]	P/LP, P/LP	Ataxia UMN features myoclonic jerks	NA	Yes	NA	NA	NA
M	24	38	No	c.132dupA [p.Asp45fs] (HOM)	P/LP	Ataxia UMN features	NA	NA	NA	NA	NA
Yang et al. (2020) [59]											
F	37	41	No	c.1244C > G [p.Ser415*] (HOM)	not reported	Gait instability, trunk and limb ataxia dysarthria horizontal gaze-evoked and downbeat nystagmus hypometric saccades brisk DTR Hoffman’s sign, Babinski sign ankle clonus	NA	No (MoCA score = 27)	Cerebellar atrophy	No	Normal
Kim et al. (2020) [60]											
M	37	45	Yes	c.1244C > G [p.Ser415*]	not reported	Ataxia oculomotor abnormalities—downbeat nystagmus pyramidal signs spasticity	NA	No	Cerebellar atrophy	No	NA
M	35	42	Yes	c.132dupA [p.Asp45fs]	P/LP	Ataxia oculomotor abnormalities—downbeat nystagmus pyramidal signs spasticity	NA	No	Cerebellar atrophy	No	NA
Nanetti et al. (2019*)* [55]											
F	51	58	Yes	c.289delA [p.Thr96_Met97ins*] (HOM)	P/LP	Dysarthria, dysmetria, gait ataxia horizontal nystagmus dysphagia increased DTR ankle clonus LL spasticity bradykinesia urinary incontinence giant SEPs tremor	9	Executive COG	Cerebellar atrophy, cortical atrophy	Epileptic spikes on EEG	LMN involvement
F	53	62	Yes	c.289delA [p.Thr96_Met97ins*] (HOM)	P/LP	Dysarthria, dysmetria, gait ataxia horizontal nystagmus dysphagia increased DTR ankle clonus LL spasticity Babinski sign bradykinesia giant SEPs tremor	10	Cognitive decline	Cerebellar atrophy, cortical atrophy	Epileptic spikes on EEG	NA
M	40	50	Yes	c.1088_1093delinsTCCTT [p.Ser363Ilefs*7] (HOM)	LP	Dysarthria, dysmetria, gait ataxia vertical ophthalmoparesis dysphagia increased DTR pes cavus bradykinesia	10	Executive COG	Cerebellar atrophy	No	Normal
F	41	54	Yes	c.1088_1093delinsTCCTT [p.Ser363Ilefs*7] (HOM)	LP	Dysarthria, dysmetria, gait ataxia horizontal nystagmus vertical ophthalmoparesis dysphagia increased DTR Babinski sign pes cavus bradykinesia	15	Executive COG	Cerebellar atrophy	Epileptic spikes on EEG	LMN involvement
F	38	62	Yes	c.289delA [p.Thr96_Met97ins*], c.518delT [p.Leu173Argfs*7]	P/LP, not reported	Dysarthria, dysmetria, gait ataxia horizontal and vertical nystagmus vertical ophthalmoparesis dysphagia increased DTR ankle clonus LL spasticity Babinski sign bradykinesia urinary incontinence tremor	17.5	Cognitive decline	Cerebellar atrophy, cortical atrophy	No	LMN involvement
M	41	47	Yes	c.1418delA [p.Asp473Alafs*36], c.337 + 1G > A	Not reported, P	Dysarthria, dysmetria, gait ataxia horizontal nystagmus increased DTR pes cavus bradykinesia	16.5	Executive COG	Cerebellar atrophy, mild dentate nuclei T2 hyperintensities, left acoustic neurinoma	No	Normal
M	33	55	Yes	c.(1797 + 1_17981)_(1913 + 1_1914-1)del [p.His600_Glu638del], c.815G > C [p.Trp272Ser]	Not reported, not reported	Dysarthria, dysmetria, gait ataxia horizontal nystagmus vertical ophthalmoparesis strabismus dysphagia increased DTR giant SEPs tremor	20	Executive COG	Cerebellar atrophy	No	LMN involvement
F	27	31	Yes	c.132dupA [p.Asp45fs], c.1291C > T [p.Glu431*]	P/LP, not reported	Dysarthria, dysmetria, gait ataxia horizontal nystagmus increased DTR pes cavus	8	Executive COG	Cerebellar Atrophy	No	Normal
F	40	70	Yes	c.1418delA [p.Asp473Alafs*36], c.1664G > C [p.Trp555Ser]	Not reported, not reported	Dysarthria, dysmetria, gait ataxia horizontal nystagmus vertical opthalmoparesis increased DTR ankle clonus LL spasticity Babinski sign bradykinesia urinary incontinence behavioral abnormalities	22	Cognitive decline	Cerebellar atrophy, cortical atrophy	No	LMN involvement
M	38	41	Yes	c.(1797 + 1_17981)_(1913 + 1_1914-1)del [p.His600_Glu638del], c.1558dupG [p.Ala520Glyfs*7]	Not reported, not reported	Dysarthria, dysmetria, gait ataxia horizontal nystagmus LL spasticity	9	NA	Cerebellar atrophy	No	NA
Sun et al. (2019) [70]											
F	NA	68	Yes	c.132dupA [p.Asp45fs] (HOM)	P/LP	Ataxia, dysarthria	NA	NA	Cerebellar atrophy	NA	NA
M	17	63	Yes	c.132dupA [p.Asp45fs] (HOM)	P/LP	Ataxia, nystagmus	NA	NA	Cerebellar atrophy	NA	NA
F	20s	57	Yes	c.96del [p.Glu33Asnfs*3], c.306C > A [p.Tyr102*]	P/LP, P	Ataxia, dysarthria dysdiadochokinesia nystagmus muscle weakness	NA	NA	Cerebellar atrophy	NA	NA
Nieto et al. (2019) [43]											
M	28	43	Yes	c.132dupA [p.Asp45fs] (HOM)	P/LP	Ataxia dysarthria nystagmus brisk reflexes	12	Executive COG (prefrontal dysfunction) borderline FIQ	Cerebellar atrophy	NA	NA
F	30	51	Yes	c.132dupA [p.Asp45fs] (HOM)	P/LP	Ataxia dysarthria nystagmus brisk reflexes restless leg syndrome	20	Yes (widespread cerebral dysfunction) extremely low FIQ	Cerebellar atrophy	NA	Normal
F	37	55	Yes	c.132dupA [p.Asp45fs] (HOM)	P/LP	Ataxia dysarthria nystagmus brisk reflexes restless leg syndrome	24	Yes (widespread cerebral dysfunction) extremely low FIQ	Cerebellar atrophy	NA	Mild axonal sensory neuropathy
Kang et al. (2019) [71]											
M	41	68	No	c.1A > T [p.0?] (HOM)	Not reported	Gait ataxia dysphagia dysarthria vertical nystagmus LL hyperreflexia urinary incontinence	20.5	NA	Severe cerebellar atrophy	NA	NA
F	32	49	No	c.132dupA [p.Asp45fs], c.1219-1G > T	P/LP, not reported	Gait ataxia UL and LL limb ataxia dysarthria horizontal nystagmus LL hyperreflexia	20	NA	Severe cerebellar atrophy	NA	NA
Coutelier et al. (2018) [69]											
NA	24	NA	NA	c.132dupA [p.Asp45fs], c.1537T>C [p.Cys513Ar]	P/LP, LP	Sensory polyneuropathy, AOA-like phenotype	NA	NA	NA	NA	NA
NA	22	NA	NA	c.132dupA [p.Asp45fs] (HOM)	P/LP	Mild spastic ataxic phenotype (stage 1 disability with no functional handicap)	NA	NA	NA	NA	NA
NA	NA	NA	NA	c.132dupA [p.Asp45fs] (HOM)	P/LP	Spastic ataxia	NA	NA	NA	NA	NA
NA	NA	NA	NA	c.289delA [p.Thr96_Met97ins*] (HOM)	P /LP	Spastic ataxia	NA	NA	NA	NA	NA
Bogdanova-Mihaylova et al. (2017) [53]											
F	43	61	Yes	c.132dupA [p.Asp45fs] (HOM)	P/LP	Gait ataxia, dysarthria, appendicular dysmetria hypermetric saccades downbeat nystagmus horizontal and vertical gaze-evoked nystagmus conjunctival and scleral telangiectasia and tortuosity increased UL and LL tendon reflexes Babinski sign LL spasticity detrusor overactivity	18	Yes	Diffuse cerebellar atrophy, frontal atrophy	No	NA
M	31	47	Yes	c.132dupA [p.Asp45fs] (HOM)	P/LP	Gait ataxia, dysarthria, appendicular dysmetria hypometric saccades downbeat nystagmus horizontal gaze-evoked nystagmus conjunctival and scleral telangiectasia and tortuosity increased LL tendon reflexes LL spasticity detrusor overactivity	9.5	Yes	Diffuse cerebellar atrophy	Single	NA
M	35	44	Yes	c.132dupA [p.Asp45fs] (HOM)	P/LP	Gait ataxia, dysarthria, appendicular dysmetria hypermetric saccades horizontal gaze-evoked nystagmus increased LL tendon reflexes unilateral Babinski sign LL spasticity distal LL amyotrophy	9.5	Yes	Diffuse cerebellar atrophy	No	NA
Bodranghien et al. (2017) [21]											
F	24	33	No	c.1219-1G > T (HOM)	not reported	Stance and gait ataxia dysmetria increased DTR gaze-evoked nystagmus hypermetric saccades scanning speech postural and kinetic tremor	NA	NA	Diffuse cerebellar cortical atrophy	No	NA
Chamard et al. (2016) [44]											
F	26	39	Yes	c.132dupA [p.Asp45fs], c.1009T>G [p.Phe337Val]	P/LP, P/LP	Gait and speech disorder limb ataxia dysarthria episodic diplopia gaze-evoked nystagmus slow saccades increased DTR mild LL spasticity	NA	Mild executive syndrome with attention impairment	Major diffuse cerebellar atrophy	Partial complex seizures with generalization	Normal
M	30	37	Yes	c.132dupA [p.Asp45fs], c.1009 T > G [p.Phe337Val]	P/LP, P/LP	Gait and speech disorder limb ataxia dysarthria gaze-evoked nystagmus slow saccades and bilateral ptosis right corticospinal hemiparesis and homonymous hemianopsia	NA	Executive syndrome with attention impairment	Cerebellar atrophy and left temporo-parieto-occipital porencephalic cyst	Partial seizures with occasional secondary tonico-clonic generalization	Normal
Mišković et al. (2016) [51]											
F	18	48	Yes	c.1150_1151del [p.Leu384fs] (HOM)	P	Gait and limb ataxia dysarthria downbeat nystagmus increased DTR Babinski sign	NA	NA	NA	No	LMN involvement
F	18	54	Yes	c.1150_1151del [p.Leu384fs] (HOM)	P	Gait and limb ataxia dysarthria downbeat nystagmus increased DTR Babinski sign	NA	Cognitive decline	NA	No	NA
M	18	58	Yes	c.1150_1151del [p.Leu384fs] (HOM)	P	Gait and limb ataxia dysarthria horizontal nystagmus increased DTR Babinski sign	NA	NA	NA	No	NA
M	18	53	Yes	c.1150_1151del [p.Leu384fs] (HOM)	P	Gait and limb ataxia dysarthria downbeat nystagmus macular degeneration increased DTR Babinski sign muscle hypotrophy and fasciculations	NA	Cognitive decline	Severe cerebellar atrophy and mild pontine atrophy	No	LMN involvement
F	6	36	Yes	c.1150_1151del [p.Leu384fs] (HOM)	P	Gait and limb ataxia dysarthria horizontal nystagmus increased DTR Babinski sign	NA	Cognitive decline	Severe cerebellar atrophy	No	LMN involvement
F	18	40	Yes	c.1150_1151del [p.Leu384fs] (HOM)	P	Gait and limb ataxia dysarthria horizontal nystagmus increased DTR Babinski sign fasciculations	NA	Cognitive decline	Severe cerebellar atrophy	No	LMN involvement
M	20	48	Yes	c.1150_1151del [p.Leu384fs] (HOM)	P	Gait and limb ataxia dysarthria horizontal nystagmus increased DTR Babinski sign	NA	Cognitive decline	Severe cerebellar atrophy	No	LMN involvement
F	26	42	Yes	c.1150_1151del [p.Leu384fs] (HOM)	P	Gait and limb ataxia dysarthria horizontal nystagmus increased DTR	NA	Cognitive decline	Severe cerebellar atrophy	No	LMN involvement
M	30	65	Yes	c.1150_1151del [p.Leu384fs] (HOM)	P	Gait and limb ataxia dysarthria downbeat nystagmus increased DTR Babinski sign muscle hypotrophy and fasciculations	NA	NA	NA	No	NA
F	20	40	Yes	c.1150_1151del [p.Leu384fs] (HOM)	P	Gait and limb ataxia dysarthria downbeat nystagmus increased DTR Babinski sign	NA	NA	NA	No	NA
M	16	36	Yes	c.1150_1151del [p.Leu384fs] (HOM)	P	Gait and limb ataxia dysarthria horizontal nystagmus increased DTR muscle hypotrophy and fasciculations	NA	NA	Severe cerebellar atrophy	No	NA
Minnerop et al. (2015) [72]											
F	20	NA	Yes	c.132dupA [p.Asp45fs] (HOM)	P/LP	Gait and stance ataxia dysmetria tension headache and rotational vertigo nystagmus saccadic pursuit bradykinesia distal LL atrophy UL intention tremor	5 (8 at the age of 24)	No	Cerebellar atrophy	No	Normal
Yoshida et al. (2014) [57]											
M	41	66	No	c.493_494dup [p.Ile166Alafs*3] (HOM)	Not reported	Gait ataxia dysarthria severe truncal ataxia increased DTR Babinski sign muscular wasting and decreased vibration sense on lower extremities	NA	No	Cerebellar atrophy	No	NA
Fogel et al. (2014) [73]											
M	NA	51	Yes	c.123_124insA [p.Asp45Argfs*9]	Not reported	Pure cerebellar ataxia	NA	NA	NA	No	NA
Balreira et al. (2014) [22]											
F	7	57	No	c.132_133insT [p.Asp45Argfs], c.1843G > A [p.Asp615Asn]	Not reported, UN	Dysarthria, dysmetria gait, limb and trunk ataxia slow saccades and saccadic pursuit low CoQ10 in muscle	NA	Cognitive dysfunction	Parieto-occipital and cerebellar atrophy	Generalized epilepsy at age of 7	Normal
F	30	52	No	c.132_133insT [p.Asp45Argfs], c.1315G > T [p.Glu382*]	Not reported, not reported	Dysarthria, dysmetria gait and limb ataxia oscilloscopia retinal fibrosis, cataracts, vitreous fluid opacity, macular degeneration downbeat nystagmus increased DTR Hoffman’s sign ankle clonus type II fiber atrophy low CoQ10 in blood and CSF	NA	No	Marked cerebellar atrophy	No	Normal
Renaud et al. (2014) [41]											
F	17	23	NA	c.1668 + 1G > A (HOM)	Not reported	Ataxia, dysarthria gaze-evoked nystagmus hypermetric saccades increased DTR	11	No	Diffuse cerebellar atrophy	No	Normal
M	33	44	NA	c.685G > T [p.Gly229Trp], c.1291C > T [p.Gln431*]	Not reported, not reported	Gait ataxia gaze-evoked nystagmus increased DTR Babinski sign left hypoacousia	14	No	Diffuse cerebellar atrophy	No	Normal
M	40	47	NA	c.1009T>C [p.Phe337Val], c.132dupA [p.Asp45fs]	P/LP, P/LP	Gait ataxia vertical nystagmus increased DTR LL spasticity	NA	No	Diffuse cerebellar atrophy	No	Motor neuron involvement in tibialis interior
F	37	43	NA	c.1214delT [p.Leu405*], c.1476 + 1G > T	Not reported, P	Gait ataxia gaze-evoked nystagmus increased DTR tongue fasciculations	6.5	No	Cerebellar atrophy	No	Normal
F	30	33	NA	del ex.12 (HOM)	Not reported	Gait ataxia hypermetric saccades increased DTR ankle clonus	10	No	Moderate cerebellar atrophy	No	NA
F	32	59	NA	c.1009T>G [p.Phe337Val] c.132dupA [p.Asp45fs]	P/LP, P/LP	Ataxia gaze-evoked nystagmus diplopia dysphagia increased DTR	NA	No	Cerebellar atrophy	No	Normal
M	43	68	NA	del ex.12 (HOM)	Not reported	Ataxia with vertigo downbeat nystagmus slow saccades stridor anxiety	NA	MMSE score = 19–29	Cerebellar atrophy	No	NA
F	33	52	NA	del ex.12, c.132dupA [p.Asp45fs]	Not reported, P/LP	Ataxia Increased DTR Babinski sign vertical and horizontal nystagmus	NA	mild intellectual disability	Cerebellar atrophy	No	NA
F	32	37	NA	c.512T>C [p.Phe171Ser], c.132dupA [p.Asp45fs]	UN, P/LP	Ataxia saccadic pursuit increased DTR LL spasticity paroxysmal limb weakness Babinski sign vertigo, cephalalgia	10.5	No	Cerebellar atrophy	No	Normal
Maruyama et al. (2014) [56]											
M	42	58	NA	c.609C > G [p.Tyr203*] (HOM)	Not reported	Cerebellar ataxia dysarthria saccadic eye movement increased DTR decreased vibration sense constipation	NA	MMSE score = 29	Mild cerebellar atrophy, slight brainstem atrophy	Episodic loss of consciousness	Normal
Chamova et al. (2012) [50]											
M	16	35	Yes	c.1150_1151del [p.Leu384fs] (HOM)	P	Dysarthria, dysmetria, gait and appendicular ataxia horizontal and vertical nystagmus tortuosity of retinal vessels increased DTR macular hypoplasia	NA	Wide-spread deficits across most cognitive domains	NA	No	NA
F	17	32	Yes	c.1150_1151del [p.Leu384fs] (HOM)	P	Dysarthria, dysmetria, gait and appendicular ataxia downbeat nystagmus increased DTR Babinski sign	NA	Wide-spread deficits across most cognitive domains	Severe diffuse cerebellar atrophy, T2/FLAIR hyperintense and T1 hypointense zones in cerebellar hemispheres	No	Motor neuron involvement in m.quadriceps femoris
M	17	29	Yes	c.1150_1151del [p.Leu384fs] (HOM)	P	Dysarthria, dysmetria, gait and appendicular ataxia downbeat nystagmus hypermetric saccades increased DTR	NA	Wide-spread deficits across most cognitive domains	NA	No	Normal
Vermeer et al. (2010) [20]											
M	25	50	Yes	c.1529T>G [p.Leu510Arg] (HOM)	P	Dysarthria, gait and appendicular ataxia downbeat nystagmus hypermetric saccades increased DTR Babinski sign cold and blue toes	NA	No	Severe cerebellar atrophy	No	Motor neuron involvement
M	20	48	Yes	c.1529T>G [p.Leu510Arg] (HOM)	P	Dysarthria, gait and appendicular ataxia downbeat nystagmus hypermetric and slow vertical saccades increased DTR wasting and fasciculations proximal leg muscles, cold and blue fingers and toes	NA	No	Severe cerebellar atrophy	No	Motor neuron involvement
F	32	47	Yes	c.1529T>G [p.Leu510Arg] (HOM)	P	Dysarthria, gait and appendicular ataxia downbeat nystagmus hypermetric saccades increased DTR cold and blue fingers and toes	NA	No	Severe cerebellar atrophy	No	NA
F	15	42	Yes	c.1150_1151del [p.Leu384fs] (HOM)	P	Dysarthria, gait and appendicular ataxia horizontal and vertical nystagmus hypermetric saccades tortuosity of conjunctival vessels increased DTR inspiratory stridor	NA	Mild intellectual disability	Severe cerebellar atrophy	No	NA
F	15	39	Yes	c.1150_1151del [p.Leu384fs] (HOM)	P	Dysarthria, gait and appendicular ataxia horizontal and vertical nystagmus tortuosity of conjunctival vessels increased DTR pes cavus	NA	Moderate intellectual disability	Severe cerebellar atrophy	No	NA
M	13	35	Yes	c.1150_1151del [p.Leu384fs] (HOM)	P	Dysarthria, gait and appendicular ataxia horizontal nystagmus hypermetric saccades tortuosity of conjunctival vessels increased DTR fasciculations leg muscles, inspiratory stridor and vocal cord paresis	NA	No	Severe cerebellar atrophy	No	Motor neuron Involvement
F	45	57	Yes	c.1476 + 1G > T, c.1604del [p.Leu535*]	P, P	Dysarthria, gait and appendicular ataxia nystagmus saccadic pursuit increased DTR mild LL spasticity slight rest tremor pes cavus	NA	No	NA	No	NA
F	25	49	Yes	c.1476 + 1G > T, c.1604del [p.Leu535*]	P, P	Dysarthria, gait and appendicular ataxia multidirectional nystagmus slow saccades episodic diplopia increased DTR Babinski sign pes cavus	NA	No	Severe cerebellar atrophy	No	Normal

*AOA *Ataxia with oculomotor apraxia, *COG* cognitive impairment, *CoQ10* coenzyme Q10, *CSF* cerebrospinal fluid, *DTR* deep tendon reflexes, *EEG *electroencephalogram, *EMG* Electromyogram, *F* female, *FIQ* Full scale intellectual quotient, *HOM* homozygous, *LL* lower limb, *LMN* lower motor neurons, *LP *likely pathogenic, *M* male, *MMSE* Mini-Mental State Examination, *MoCA* Montreal Cognitive Assessment, *MRI* magnetic resonance image, *NA* not available, *P* pathogenic, *PP* predicted pathogenicity, *SARA* Scale for Assessment and Rating of Ataxia, *SEPs* sensory evoked potentials, *UMN* upper motor neurons,* UL* upper limb, *UN* uncertain

#### ANO10 Variants Associated with Other Conditions and Characteristics

*ANO10* variants are linked to other conditions as well. SNPs in the gene were reported to be associated with left-handedness, schizophrenia [61], and tiredness [62]. *ANO10* rs118005571 correlated with a decreased risk of biochemical recurrence following radical prostatectomy, even though no significant association between ANO10 expression and prostate cancer occurred [63]. On the other hand, SNP rs41289586, corresponding to the missense variant c.788G>A [p.Arg263His], was identified through GWAS as a risk factor for developing primary central nervous system lymphoma [64]. The same variant has been shown to be involved in innate immune defence against *Borrelia* infection. Specifically, the p.Arg263His variant failed to generate Cl^−^ currents in macrophages, thus compromising their migration and ability to eliminate *Borrelia* spirochetes. This finding established ANO10 as a novel player in host defence [24]. *ANO10* upregulation in hepatocellular carcinoma, and association with poor prognosis, further suggest an involvement in the immune defence. ANO10 presumably regulates the immune microenvironment of the tumour [65]. Interestingly, in vitro *Ehrlichia ruminantium* infection of bovine aorta endothelial cells revealed ANO10 overexpression suggesting a significant impact of ANO10 on endothelial inflammatory responses and vascular permeability [66].

## Discussion

Anoctamin family members show functional duality; they function as ion channels or lipid scramblases, activated by calcium binding in either case. The family members displaying channel activity adopt only ion-conductive conformation, while those exhibiting scrambling activity can adopt an additional, lipid-conductive conformation, as well. This difference is attributed to small conformational changes in the permeation pathway of the protein. Remarkably, point mutations can switch the function of the protein from ion to lipid permeation and vice versa. An increase in the intracellular Ca^2+^ concentration initiates the activation of both scramblases and channels of the anoctamin family. Calcium binding to the protein leads to a conformational rearrangement in helix α6, which in turn induces gating motions, i.e. the expansion of the pore in channels or the exposure of the subunit cavity in scramblases. Both of these activation steps can be affected by various modulators, such as ions, membrane lipids, and phosphatidylinositol 4,5-bisphosphate (PIP2) [29].

Anoctamin 10, which is the focus of the present review, exhibits the activities shared by the anoctamin family members. It is an ER-residing lipid scramblase with non-selective channel activity. It has been suggested that ANO10 is constantly active to ensure the even distribution of newly synthesized lipids to both leaflets of the endoplasmic reticulum [13]. Homozygous or compound heterozygous *ANO10* variants are considered causative factors for SCAR10, a rare, gradually progressive spinocerebellar ataxia. Though the molecular basis of SCAR10 remains unknown, several mechanisms implicating ANO10 in disease pathogenesis have been proposed.

Considering ANO10’s scrambling activity and that variants in *ANO10* lead to SCAR10, abnormal lipid distribution in ER and other membranes was proposed as a potential cause of ataxia [13]. Defects in endosomal transport are an additional factor that might contribute to SCAR10 pathology. ANO10 was found to regulate endosomal sorting, and the late endolysosomal pathway. Furthermore, the absence of ANO10 results in defects in endosomal sorting which can be rescued by the wild type protein but not by SCAR10-causing variants. These findings highlight ANO10 as an endolysosomal system regulator in SCAR10 aetiology [17]. However, the most prominent mechanism believed cause cerebellar ataxia is the degeneration of Purkinje cells. Degenerated or functionally altered Purkinje cells lead to the attenuation of inhibitory signals to the deep cerebellar nuclei, increased hyperexcitability of the latter, and eventually impaired motor performance [67]. Specifically, the aberrant calcium signalling in Purkinje neurons is suggested to induce the onset of cerebellar ataxia pathogenesis. Disruptions in calcium signalling result in defective synaptic neurotransmission and neuroplasticity, leading to cell death [68]. The calcium-activated chloride channel activity of ANO10 and the association between *ANO10* variants and SCAR10 development imply an important role of ANO10 in SCAR10 pathology, presumably through the regulation of calcium signalling.

## Conclusion

The present study discusses the relationship between structure and function and the possible ANO10 molecular mechanisms that induce SCAR10 pathology. The extensive phenotype characterization (including both common and rare clinical features of SCAR10) provided in the current study, and the identification of *ANO10* variants found in SCAR10 patients, will facilitate the diagnosis and may be useful for disease prognosis. Nevertheless, further investigation is imperative to unravel the precise association between the ANO10 mechanism and the development and progression of SCAR10, to offer a better insight into SCAR10 biology and assist in developing novel therapeutic approaches.

## Supplementary Information

Below is the link to the electronic supplementary material.

Supplementary file1 (DOCX 55 KB)
