# Supplementary figures and images for: ANO10 Function in Health and Disease
Source: Cerebellum. 2022 Jun 1;22(3):447–67. doi: 10.1007/s12311-022-01395-3 (PMC10126014; doi:10.1007/s12311-022-01395-3)

**
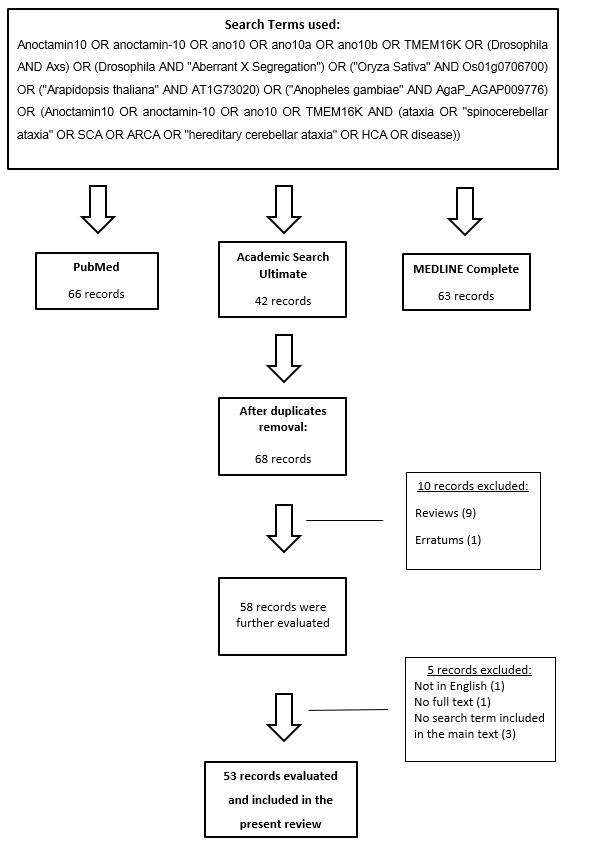
Supplementary figure 1.** Flow diagram study.

Supplement: Supplementary file 1 — Supplementary file1 (DOCX 55 KB) [file 12311_2022_1395_MOESM1_ESM.docx]
